# Frequency and phenotypic characteristics of *RPE65* mutations in the Chinese population

**DOI:** 10.1186/s13023-021-01807-3

**Published:** 2021-04-13

**Authors:** Feng-Juan Gao, Dan-Dan Wang, Jian-Kang Li, Fang-Yuan Hu, Ping Xu, Fang Chen, Yu-He Qi, Wei Liu, Wei Li, Sheng-Hai Zhang, Qing Chang, Ge-Zhi Xu, Ji-Hong Wu

**Affiliations:** 1 Eye Institute, Eye and ENT Hospital, College of Medicine, Fudan University, Shanghai, 200032 China; 2 Shanghai Key Laboratory of Visual Impairment and Restoration, Science and Technology Commission of Shanghai Municipality, Shanghai, China; 3 BGI-Shenzhen, Shenzhen, Guangdong China; 4 Key Laboratory of Myopia (Fudan University), Chinese Academy of Medical Sciences, National Health Commission, Yangpu District, China; 5 Laboratory of Genomics and Molecular Biomedicine, Department of Biology, University of Copenhagen, Copenhagen, Denmark; 6 Department of Computer Science, City University of Hong Kong, Kowloon, Hong Kong China; 7 BGI Education Center, University of Chinese Academy of Sciences, Shenzhen, China; 8 Shenzhen Engineering Laboratory for Birth Defects Screening, BGI-Shenzhen, Shenzhen, China

**Keywords:** Inherited retinal dystrophy, *RPE65* gene mutations, Next-generation sequencing, Chinese population, Genotype–phenotype correlations

## Abstract

**Background:**

The retinoid isomerohydrolase *RPE65* has received considerable attention worldwide since a successful clinical gene therapy was approved in 2017 as the first treatment for vision loss associated with *RPE65*-mediated inherited retinal disease. Identifying patients with *RPE65* mutations is a prerequisite to assessing the patients’ eligibility to receive *RPE65-*targeted gene therapies, and it is necessary to identify individuals who are most likely to benefit from gene therapies. This study aimed to investigate the *RPE65* mutations frequency in the Chinese population and to determine the genetic and clinical characteristics of these patients.

**Results:**

Only 20 patients with *RPE65* mutations were identified, and *RPE65* mutations were determined to be the 14th most common among all patients with genetic diagnoses. Ten novel variants and two hotspots associated with FAP were identified. A literature review revealed that a total of 57 patients of Chinese origin were identified with pathogenic mutations in the *RPE65* gene. The mean best Snellen corrected visual acuity was worse (mean 1.3 ± 1.3 LogMAR) in patients older than 20 years old than in those younger than 15 years old (0.68 ± 0.92 LogMAR). Bone spicule-like pigment deposits (BSLPs) were observed in six patients; they were older than those without BSLP and those with white-yellow dots. Genotype–phenotype analysis revealed that truncating variants seem to lead to a more severe clinical presentation, while best corrected visual acuity testing and fundus changes did not correlate with specific *RPE65* variants or mutation types.

**Conclusions:**

This study provides a detailed clinical-genetic assessment of patients with *RPE65* mutations of Chinese origin. These results may help to elucidate *RPE65* mutations in the Chinese population and may facilitate genetic counseling and the implementation of gene therapy in China.

**Supplementary Information:**

The online version contains supplementary material available at 10.1186/s13023-021-01807-3.

## Background

The retinoid isomerohydrolase *RPE65* has received considerable attention worldwide since a successful clinical gene therapy was approved in 2017 as the first treatment for vision loss associated with *RPE65*-mediated inherited retinal disease (IRD) (https://www.fda.gov/NewsEvents/Newsroom/PressAnnouncements/ucm589467.htm). Identifying patients with *RPE65* mutations is a prerequisite to assessing the patients’ eligibility in receiving *RPE65*-targeted gene therapies, and it is necessary to identify individuals who are most likely to benefit from gene therapies.

To date, nearly 200 disease-causing mutations in the *RPE65* gene have been reported (Human Gene Mutation Database (HGMD); professional version 2019.2), which are associated with a large heterogeneous group of retinal dystrophies, including Leber congenital amaurosis (LCA) type 2, early onset severe retinal dystrophy, retinitis pigmentosa (RP) type 20 and fundus albipunctatus (FAP). Studies have shown that the mutation frequency and phenotypic variation of *RPE65* varies notably between different ethnic groups. For example, *RPE65* mutations are thought to affect approximately 1000 to 3000 people in the United States (Population clock. 2018. Available: https://www.census.gov/popclock/ [Accessed 14 Aug 2018]), while 6% of all LCA cases in Caucasians [1], 16% in the Danish LCA cohort [2], and only a few LCA cases were reported in Chinese populations [3, 4]. Generally, most studies associated with *RPE65* mutations were performed in Western populations [2, 5, 6], and the exact frequency of *RPE65* mutations in all forms of IRD and the variety of associated phenotypes in China has not been determined.

In the current study, we performed a comprehensive mutation analysis in 1434 IRD patients. Twenty patients with *RPE65* mutations were identified, and their specific clinical phenotypes were presented. Moreover, we further reviewed the varied phenotypes and genotypes of all cases of *RPE65* mutations of Chinese origin reported in the literature. These results provide a brief overview of the frequency and phenotypic characteristics of the *RPE65* mutation in the Chinese population.

## Methods

### Subjects, ethics statement and NGS analysis

A total of 1434 Chinese patients with IRDs and their available family members (total participants: 3576) were recruited from the eye genetic disease clinic of the Eye and ENT Hospital of Fudan University between January 2017 and June 2019. Of these, 956 patients had been mentioned in our previous report [Gao and others 2019]. Written informed consent in accordance with the tenets of the Declaration of Helsinki was obtained from all participants or their guardians. This study was approved by the Ethics Committee of the Eye and ENT Hospital of Fudan University. DNA was isolated from peripheral blood using the FlexiGene DNA Kit (Qiagen, Venlo, the Netherlands) according to the manufacturer’s protocol. NGS analysis and bioinformatics analysis were performed as previously reported [7]. We designed a high-throughput targeted enrichment approach to exon-capture regions of 586 genes that are involved in common inherited eye diseases. The probe length of the panel is 90 nt, the total target area obtained is 2.3 M. On average, the mean coverage depth was more than 400X, and the coverage of target region was ~ 99.9% using BGISEQ-2000. Then the sequence data obtained were analyzed as previously reported [7]. Previous reported variants were determined using Human Gene Mutation Database (HGMD, professional version 2019.2). For variants that passed the initial filtration, Sanger sequencing was carried out for segregation analysis and variants validation.

### Clinical evaluations

All patients with pathogenic mutations in *RPE65* underwent a full ophthalmic examination, including best Snellen corrected visual acuity testing (BCVA, they were converted to equivalent value of logarithm of minimal angle of resolution (logMAR) unit), slit lamp biomicroscopy, tonometry, fundus examination, wide-field fundus imaging (Optos PLC, Dunfermline, United Kingdom), swept-domain optical coherence tomography (SD-OCT, Spectralis HRA + OCT, Heidelberg, Engineering Inc., Heidelberg, Germany), visual field (Humphrey Visual Field Analyzer, Carl Zeiss Inc., CA, USA), and full-field electroretinography (ERG, according to the standards of the International Society for Clinical Electrophysiology of Vision; available at www.iscev.org).

## Results

### Genetic analyses

Of the 1434 patients with IRD, 74.55% of patients (n = 1069) received a genetic diagnosis, and a total of 1516 variants involved 87 genes were identified. Only 41 alleles representing 26 distinct variants in 17 families (20 patients: 10 males, 10 females) were identified in the *RPE65* gene (NM_000329.2, Table 1 and Additional file 5: Table S1), accounting for 2.83% of all the variants, and the gene was ranked as the seventh most common gene detected in this cohort of patients with IRD (Additional file 1: Figure S1A and S1B). However, the number of patients with *RPE65* mutations only accounted for 1.87% (20/1069) of all patients with genetic diagnoses and was the 14th most common among all the patients (Additional file 1: Fig. 1c, d). Pedigrees and mutations of the 17 families are available in Additional file 2: Figure S2. Of the 26 distinct variants identified in this study, 10 (c.1039C>T p.Arg347Cys, c.1255C>T p.Pro419Ser, c.1444G>A p.Asp482Asn, c.334T>A p.Cys112Ser, c.354-2A>G, c.376del p.Val126*fs1, c.806_809delinsTGGAGCCATGAAG p.SerLeu269MetGluProTer, c.837del p.Phe279Leufs46, c.886del p.Arg296*fs1, c.94+2T>A) were novel (Additional file 3: Figure S3), including seven likely pathogenic variants and three missense variants of uncertain significance (p.Pro419Ser, p.Cys112Ser and p.Arg347Cys), which are localized in highly conserved residues (Additional file 4: Figure S4A). Bioinformatics analysis results of the novel variants are shown in Additional file 6: Table S2. Of the 16 variants reported previously, p.His68Tyr and c.998+1G>A were firstly reported to be associated with LCA.

**Table 1:** *RPE65* variants identified in this cohort of patients

Nucleotide change	Amino acid change	Mutation type	Exon/intron	Patients	ACMG category	References
c.1399C>G	p.Pro467Ala	Missense	E13	F1-1, F3-1	P	[8, 13]
c.272G>A	p.Arg91Gln	Missense	E4	F2-1	P	[14–16]
c.271C>T	p.Arg91Trp	Missense	E4	F2-1, F16-1	P	[17, 18]
c.1338G>T	p.Arg446Ser	Missense	E12	F5-1, F7-1, F7-2	P	[4, 19]
c.1543C>T	p.Arg515Trp	Missense	E14	F6-1, F6-2	P	[20, 21]
c.1444G>A	p.Asp482Asn	Missense	E13	F6-1, F6-2, F17-1	LP	Novel
c.1255C>T	p.Pro419Ser	Missense	E12	F8-1	VUS	Novel
c.202C>T	p.His68Tyr	Missense	E3	F8-1	P	[22, 23]
c.1590C>A	p.Phe530Leu	Missense	E14	F9-1	P	[4, 24]
c.997G>C	p.Gly333Arg	Missense	E9	F10-1	P	[25]
c.334T>A	p.Cys112Ser	Missense	E4	F10-1	VUS	Novel
c.131G>A	p.Arg44Gln	Missense	E3	F12-1	P	[14, 26]
c.200T>G	p.Leu67Arg	Missense	E3	F13-1	P	[27, 28]
c.1304A>G	p.Tyr435Cys	Missense	E12	F13-1	P	[29]
c.1039C>T	p.Arg347Cys	Missense	E10	F14-1	VUS	Novel
c.1078G>C	p.Ala360Pro	Missense	E10	F14-1	LP	[30]
c.493C>T	p.Gln165*	Nonsense	E5	F4-1, F4-2	P	[4]
c.1380G>A	p.Trp460*	Nonsense	E13	F15-1	P	[31]
c.94+2T>A	-	Splicing	I2	F1-1	LP	Novel
c.998+1G>A	-	Splicing	I10	F3-1	P	[13]
c.354-2A>G	-	Splicing	I5	F7-1, F7-2	LP	Novel
c.858+1del	-	Splicing	I9	F9-1, F12-1	P	[8]
c.376del	p.Val126fs*1	Frameshift	EX5	F16-1	LP	Novel
c.806_809delinsTGGAGCCATGAAG	p.SerLeu269MetGluProTer	Frameshift	EX8	F17-1	P	Novel
c.837del	p.Phe279Leufs*46	Frameshift	EX8	F11-1	LP	Novel
c.886del	p.Arg296fs	Frameshift	EX9	F15-1	LP	Novel

F: family; E: Exon; I: Intron; P: Pathogenic; LP: Likely pathogenic; VUS: variants of uncertain significance

**Fig. 1:**
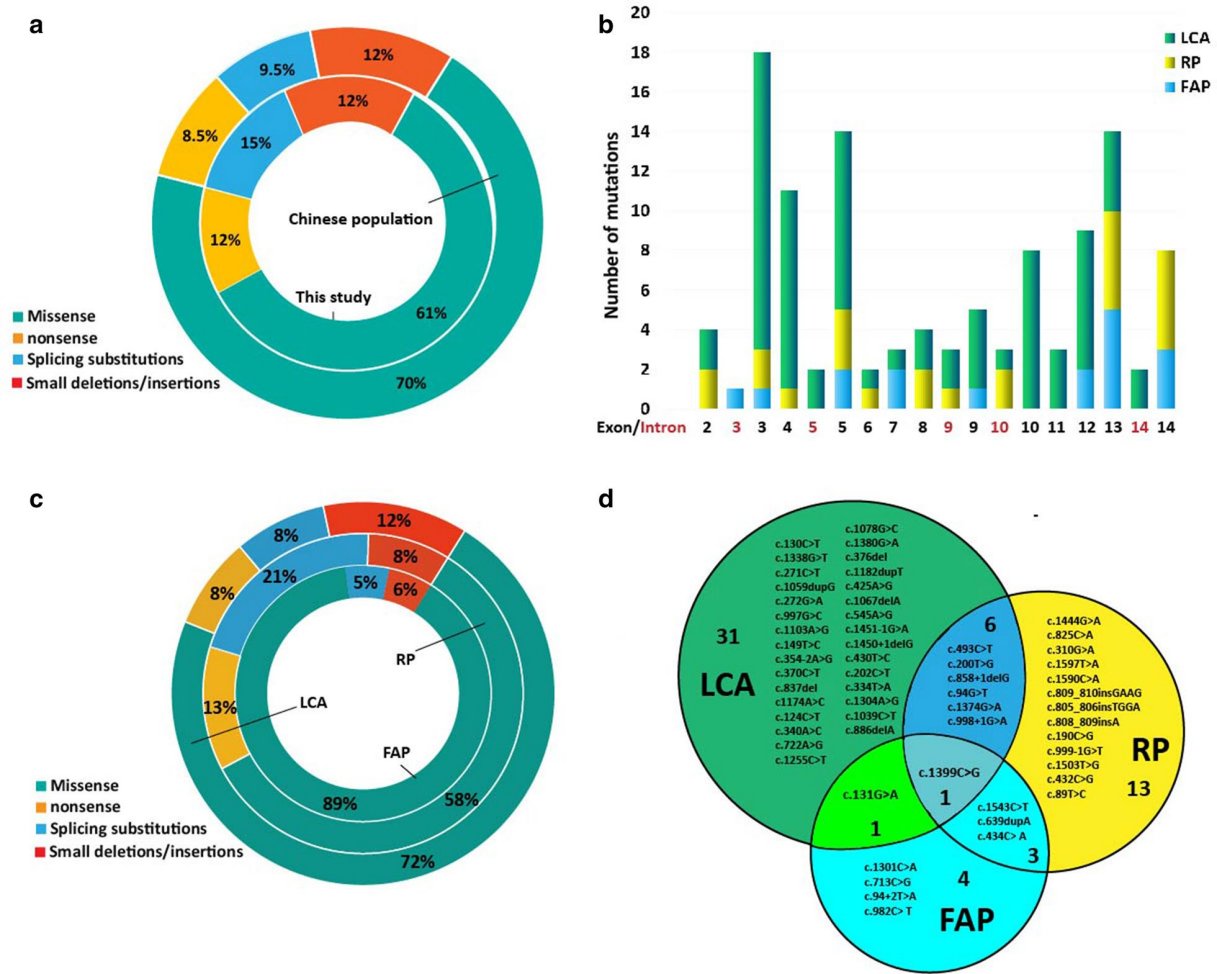
**a** Distribution of different types of variants in the *RPE65* identified in this study and reported in the Chinese population. **b** Distribution of the *RPE65* mutations reported in the Chinese population. The number of mutations located in different exons is marked in black font, and those in introns are marked in red font. **c** Distribution of different types of variants in the *RPE65* identified in LCA, RP, and FAP patients reported in the Chinese population. **d**. *RPE65* variants associated with LCA, RP and FAP. A total of 39 variants have been reported to be associated with LCA, 23 with RP, and 8 with FAP. p.Pro467Ala was associated with LCA, RP and FAP, p.Arg44Gln was associated with both LCA and FAP, six variants were associated with both RP and LCA, and three variants were associated with both RP and FAP. LCA: leber congenital amaurosis; RP: retinitis pigmentosa; FAP: fundus albipunctatus

To date, a total of 39 patients from 27 unrelated Chinese families have been reported with pathogenic mutations in *RPE65* (Additional file 5: Table S1). Together with the 20 patients (two have been previously reported: F4-1 and F5-1) in this study [8], 57 patients of Chinese origin were diagnosed. Of the 115 variants identified in these patients, the majority of pathogenic defects (71.3%, n = 82) were missense variants, and 28.7% (n = 33) were nonsense, frameshift, or splice-site mutations that severely affected protein function (Fig. 1a). Variants were distributed from exon 2 to exon 14 (Fig. 1b).

### Phenotypic characterization

**Fig. 2:**
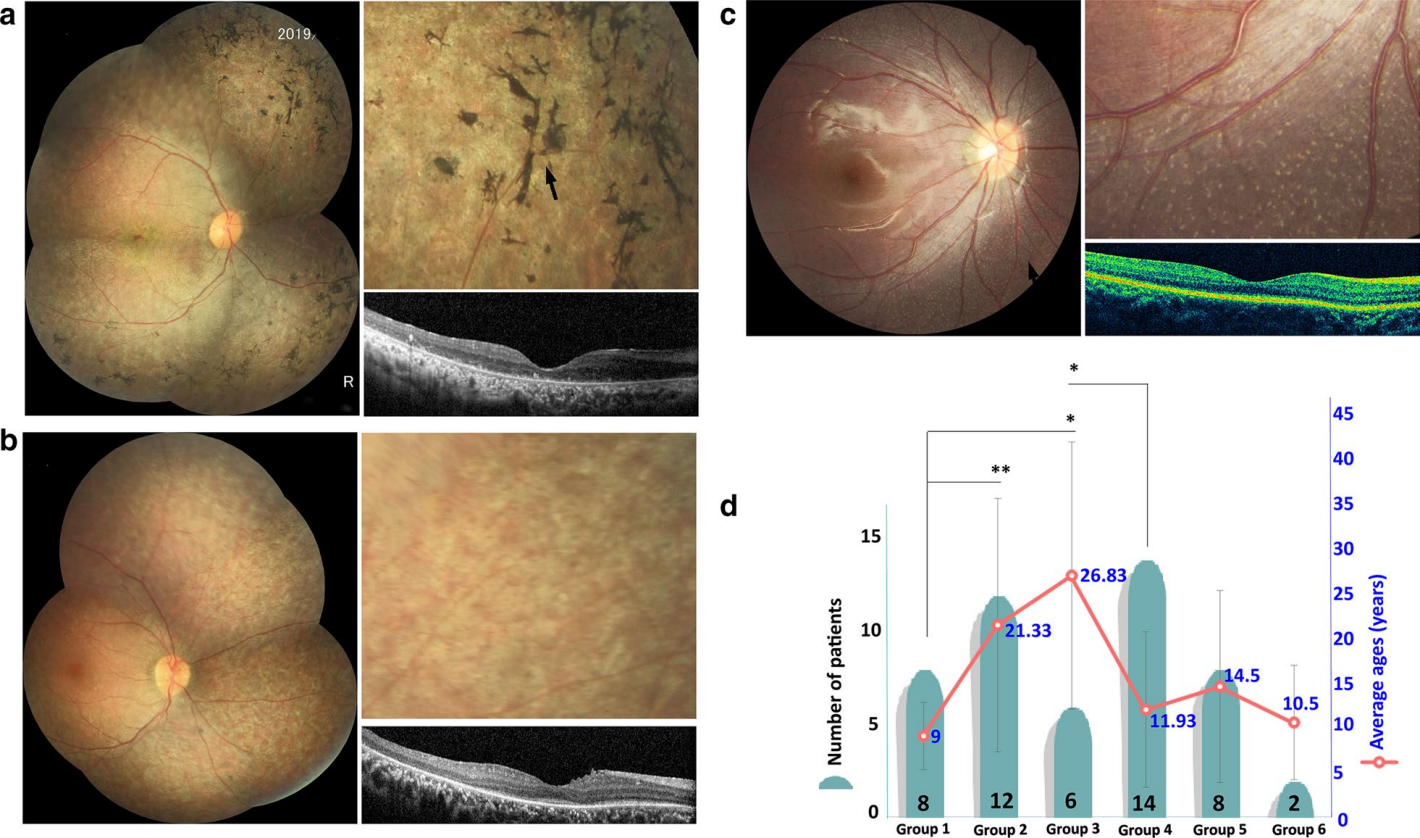
**a** Color fundus photographs and spectral domain optical coherence tomography (SD-OCT) of patients showed bone spicule-like pigment deposits (BSLP). The image in the upper right is the corresponding enlarged figure showing BSLP in the fundus (black arrow). **b** Color fundus photograph and SD-OCT of patients without BDLP or white-yellow dots (WYD). The image in the upper right is the corresponding enlarged figure showing pigment dispersion in the mid-periphery. **c** Color fundus photograph and SD-OCT of patients with WYD (black arrow). The image in the upper right is the corresponding enlarged figure showing WYD scattered in the periphery of the retina. **d** Number of patients (black font) and their corresponding mean ages (blue font) in different groups. Group 1: patients with WYD. Group 2: patients without WYD. Group 3: patients with BDLP. Group 4: patients without BDLP. Group 5: patients without WYD and BDLP. Group 6: patients with WYD and BDLP. *p < 0.05; **p < 0.01

Of the 20 patients in the 17 families, four were diagnosed with RP, one with FAP, and 15 with LCA (Table 2). The mean age at visit was 16.4 ± 12.59 years (range 3–49 years; median, 10 years). All accepted patients (LCA and RP) experienced poor vision at an early age. The mean BCVA with LCA patients was 0.82 ± 0.92 (range 3.00–0.40) LogMAR, and 90% (27/30) of eyes had a BCVA worse than 0.52 LogMAR. Of patients younger than 15 years, the mean BCVA was 0.68 ± 0.92 (range 1.30–0.40) LogMAR, while for patients older than 20 years, the mean BCVA was worse (p < 0.001, mean 1.30 ± 1.30 (range 3.00–1.00) LogMAR. Of the four patients with RP, the mean BCVA was 0.37 ± 1.05 (range 0.52–0.22) LogMAR, while the FAP patient maintained better vision (0/0.05 LogMAR). Bone spicule-like pigment deposits (BSLPs) were observed in six patients from five families (30%, 6/20, Fig. 2a), and no typical deposits were seen in 70% of patients (n = 14, Fig. 2b). Interestingly, we observed that subjects with BSLP were older than those without BSLP (p < 0.05, 26.83 ± 14.86 vs. 11.93 ± 8.65 years, respectively) (Fig. 2d). Eight patients (40%, mean age 9.0 ± 3.74 years) showed white-yellow dots (WYD) scattered in the periphery of the retina (n = 5) or perimacular area (n = 3, Fig. 2c); they were younger than those (n = 12, mean age 21.33 ± 14.09 years) without WYD (p < 0.01) and younger than patients with BSLP (p < 0.05, Fig. 2d). Cone and rod responses on ERG were nonrecordable in 14 of the 15 LCA patients and notably attenuated in the four RP patients.

**Table 2:** Clinical characteristics of the 20 patients identified in this study

Patients	Age (years) /sex	BCVA LogMAR R/L	Refraction R/L	Age at disease presentation (years)	ERG	Fundus	Others	Diagnosis
F1-1	12/F	0/0.05	+ 0.5/ + 0.75	Congenital	Undetectable rod ERG, subnormal cone ERG	a	No	FAP
F2-1	6/M	0.60/0.82	− 0.5/− 2.0	Congenital	Extinct	a, b	Nystagmus	LCA 2
F3-1	7/M	0.40/0.40	− 4.0/− 3.75	Congenital	Extinct	N	Nystagmus	LCA 2
F4-1	15/M	0.52/0.52	− 3.5/− 3.0	Congenital	Extinct	a, b	Nystagmus	LCA 2
F4-2	3/F	1.00/1.00	+ 0.5/ + 0.75	Congenital	Extinct	N	Nystagmus	LCA 2
F5-1	9/F	1.30/0.70	+ 4.5/ + 4.5	Congenital	Extinct	a	Nystagmus	LCA 2
F6-1	10/M	0.22/0.40	− / + 0.5	3	Profoundly attenuated rod and cone ERGs	N	No	RP 20
F6-2	9/F	0.40/0.40	− / + 0.25	3	Profoundly attenuated rod and cone ERGs	a	No	RP 20
F7-1	29/M	1.00/3.00	–	Congenital	Extinct	b	Nystagmus	LCA 2
F7-2	31/M	1.3/1.3	–	Congenital	Extinct	b	Nystagmus	LCA 2
F8-1	7/F	1.00/0.13	+ 1.5/ + 2.25	Congenital	Extinct	a	Nystagmus	LCA 2
F9-1	11/M	0.52/0.40	− 0.5/− 0.5	Congenital	Profoundly attenuated rod and cone ERGs	a	No	RP 20
F10-1	49/F	3.00/3.00	–	Congenital	Extinct	b	Nystagmus	LCA 2
F11-1	20/M	1.00/1.00	− 2.25/− 1.75	Congenital	Profoundly attenuated rod and cone ERGs	N	Nystagmus	LCA 2
F12-1	31/F	1.00/1.00	− 1.0/− 1.25	Congenital	Extinct	b	Nystagmus	LCA 2
F13-1	9/F	0.52/0.40	+ 2.5/ + 2.25	Congenital	Extinct	N	Nystagmus, oculo-digital sign	LCA 2
F14-1	5/F	0.60/0.70	+ 2.25/ + 3.0	Congenital	Extinct	N	Nystagmus, esotropia	LCA 2
F15-1	5/M	0.82/1.00	+ 2.0/ + 2.25	Congenital	Extinct	N	Nystagmus	LCA 2
F16-1	30/M	2.00/2.00	− /−	Congenital	Extinct	N	Nystagmus	LCA 2
F17-1	30/F	0.30/0.40	-4.0/-4.5	Congenital	Profoundly attenuated rod and cone ERGs	N	No	RP 20

F: family; R: right; L: left; LP = 3 LogMAR; HM = 2 LogMAR; a. white or white-yellow dots; b. Bone-spicule-like pigment; deposits; N: no a or b; LCA: Leber congenital amaurosis; RP: retinitis pigmentosa; FAP: fundus albipunctatus

### Genotype–phenotype correlations

**Fig. 3:**
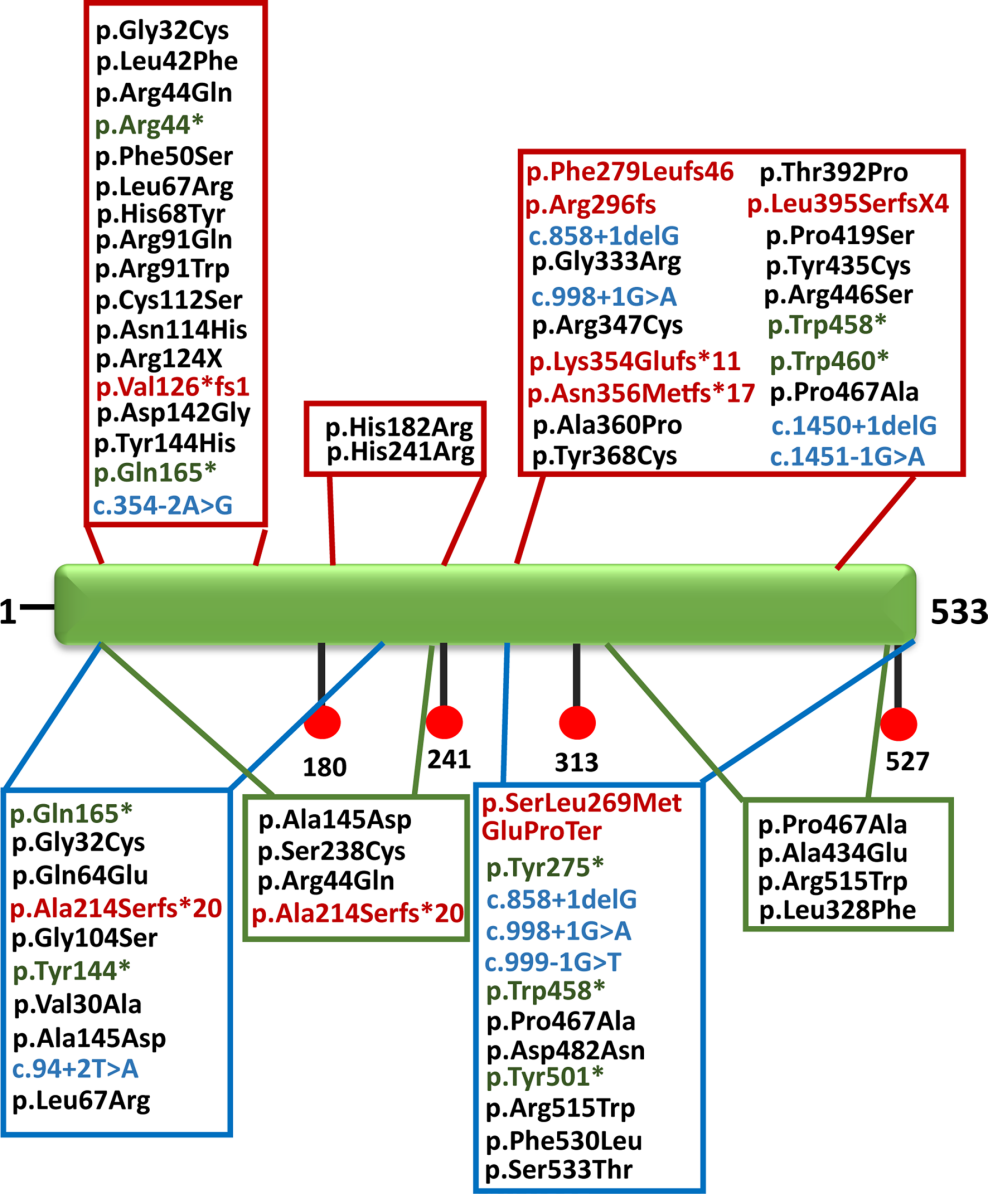
Overview of *RPE65* mutations reported to date in the Chinese population and this study. Blank: missense mutation. Blue: splicing mutation. Red: frameshift mutation. Green: nonsense mutation. Variants in the black box are mutations associated with LCA. Variants in the blue box are mutations associated with RP. Variants in the green box are mutations associated with FAP. LCA: leber congenital amaurosis; RP: retinitis pigmentosa; FAP: fundus albipunctatus

All *RPE65* variants identified in the Chinese population are shown in Fig. 3 and Additional file 5: Table S1. To date, 39 variants have been reported to be associated with LCA, 21 with RP, and 8 with FAP. As no clinical features of the FAP patient with one frameshift were provided by Guoxing Yang et al. [9], the FAP diagnosis requires further confirmation, and this patient was not included in the statistical analysis. Of all the variants associated with LCA (n = 73), 71.2% (n = 52) were missense variants, and 28.8% (n = 21) were nonsense (n = 5), frameshift (n = 9), or splice-site mutations (n = 7) that severely affected protein function, while in the FAP group (n = 16), 93.75% (n = 15) variants were missense, and only one splice-site mutation was identified (Fig. 1c). In the RP group, 58.3% (n = 14) of variants were missense variants, 20.8% (n = 5) were truncating stops, and 20.8% were frameshift (n = 2) or splicing defects (n = 3). Therefore, of all the variants (n = 97) associated with LCA and RP, which have a severe early-onset clinical presentation, only 68% (n = 66) were missense mutations, and the remaining 32% (n = 31) of variants were truncating mutations. Nevertheless, in the FAP group, which showed relatively mild symptoms, 93.8% (n = 15) of all the variants were missense. Of all the LCA and RP patients, 50% had missense + missense mutations, 35.4% had missense + nonsense/frameshift/splice-site mutations, and 14.6% did not have any missense mutations. However, of the eight FAP patients, 7 were missense + missense, and only one was a missense + splice-site mutation.

Only one variant, c.1399C>G (p. Pro467Ala), was associated with LCA, RP and FAP (Fig. 1d). This mutation was located in a highly evolutionarily conserved region (Additional file 4: Figure S4A) and altered the corresponding amino acid from proline to alanine. The 3D structural model of these amino changes is portrayed in Additional file 4: Figure S4B. Of the eight FAP patients, five had the p. Pro467Ala mutation, and the other three had the p.Arg515Trp mutation. It is likely that the two variants were hotspots of FAP. c.131G>A p.Arg44Gln was associated with both LCA and FAP; when the second mutated allele is a truncating mutation (c.858 + 1del), the patients were likely to present an LCA diagnosis, while if the second mutated allele is a missense mutation (p.Arg515Trp), the patients were likely to present an FAP diagnosis. p.Arg515Trp, p.Ala145Asp and p.Ala214Serfs*20 were associated with both RP and FAP, while p.Ala434Glu, p.Ser238Cys, c.94+2T>A, and p. Leu328Phe were only associated with FAP (Fig. 1d). Further analysis of the relationship between BCVA and fundus changes with specific mutations revealed that there was no correlation between them. Different individuals, even those with the same mutation, were found to show different changes. For example, patient F6-2 showed white-yellow dots scattered in the periphery of the retina, while patient F6-1, who had the same mutations, did not display these dots. However, we can't rule out the possibility that these dots would disappear with age.

## Discussion

*RPE65* mutation-associated IRD usually occurs at an early age, and the majority of patients become fully blind in childhood or adulthood [10, 11]. Recent research has indicated that certain forms of *RPE65*-mediated IRDs are amenable to gene therapy. Achieving an in-depth understanding of *RPE65* mutations and related phenotypic characteristics in the Chinese population is a prerequisite for developing *RPE65*-targeted gene therapies in China.

Only 20 patients with associated *RPE65* mutations were identified from January 2017 to June 2019 in our hospital, accounting for 1.87% of all IRD patients and making *RPE65* mutations the 14th most common among all patients. Together with other patients who have been reported, only 57 patients with *RPE65* mutations have been identified in China [3, 8–16]. These results suggest that *RPE65* mutations are rare in Chinese populations. As these mutations appear to be a low-probability, high-cost event, our data may provide strong clinical-based evidence for gene therapy researchers, economists, government policy makers, and ophthalmologists to make decisions in their corresponding work. The mean age of patients with *RPE65* mutations was 16.4 ± 12.59 years (median, 10 years), and the mean BCVA was 0.82 ± 0.92 LogMAR with LCA patients and 0.37 ± 1.05 LogMAR with RP patients. Of patients younger than 15 years, the mean BCVA was 0.68 ± 0.92 LogMAR, while for patients older than 20 years, the mean BCVA was worse (mean 1.3 ± 1.3 LogMAR). These data indicate that the optimal intervention window for subretinal gene therapy is within the first 2 decades of life.

El Matri, L. et al. reported that white dot deposits occurred in earlier stages, and clumped pigment occurred in later stages, in patients with *RPE65* mutations [12]. A similar result was obtained in the current study: patients with *RPE65* mutations are more likely to exhibit WYD in the first decade but show BSLP after the second decade of life. However, some patients (40%) could not have BSLP or WYD, and these changes had no correlation with specific mutations or types or locations of mutations.

To date, genotype–phenotype correlations of patients with *RPE65* mutations have not been highly distinct. It has not been determined why some mutations in *RPE65* lead to LCA, while others lead to RP or FAP. In addition, the fact that most patients are compound heterozygotes hinders efforts to assess the effect of each mutation on the phenotypes and to evaluate possible allelic hierarchy. In this study, we found four notable cases of possible discordancy between clinical and molecular diagnosis. First, truncating variants seem to lead to a more severe clinical presentation (LCA or RP), while almost all FAP patients were caused by missense mutations. Second, two hotspots (p.Pro467Ala and p.Arg515Trp) associated with FAP were identified, helping to further elucidate the mutational spectrum of *RPE65* in the Chinese population. Third, p.Pro467Ala was associated with LCA, RP and FAP, suggesting that this mutation may have a mild effect on protein function, and the phenotype is primarily affected by the second allele. Fourth, BCVA and fundus changes did not correlate with specific *RPE65* variants or mutation types.

## Conclusions

In the current study, we performed a comprehensive analysis of the phenotypes and genotypes of the 20 patients with *RPE65* mutations identified in this study and all the *RPE65* mutation cases of Chinese origin reported in the literature. Our data provide a brief overview of the frequency and phenotypic characteristics of *RPE65* mutations in the Chinese population, help to characterize *RPE65* mutations in China, and represent a possible reference for genetic counseling and the selection of eligible patients for gene augmentation.

## Supplementary Information

**Additional file 1: Figure S1**. A. Distribution of inherited retinal disease (IRD)-causative genes variants (n = 1516) in 1069 patients. *RPE65* mutations (n = 41) accounted for 2.70% of all the variants. B. Number of mutations of different IRD-causative genes. Only 41 variants were identified in the *RPE65* gene, which ranked as the seventh most commonly detected gene in this cohort of patients with IRD. C. Percentage of IRD patients with variants in different causative genes; patients with *RPE65* mutations only accounted for 1.87% of all patients (n = 1069). D. Number of IRD patients with different causative genes. Twenty patients with *RPE65* mutations were identified, and they were placed as the 14th among all the patients.

**Additional file 2: Figure S2**. Pedigrees of the 17 families with *RPE65* mutations. Filled symbols signify patients. Unfilled symbols represent unaffected family members. Arrows: probands. Square: male individuals; Circle: female individuals. A slash indicates a deceased person.

**Additional file 3: Figure S3**. Sequencing results of the ten novel *RPE65* variants identified in this study. Arrows indicate the position of the mutated nucleotide. Mutation c.806_809delinsTGGAGCCATGAAG, c.493C>T, c.354-2A>G, c.1255C>T, c.334T>A and c.1444G>A were shown in reverse strand.

**Additional file 4: Figure S4**. A. Multiple sequence alignment of different species of *RPE65* mutations (c.1255C>T p.Pro419Ser, c.334T>A p.Cys112Ser, c.1039C>T p. Arg347Cys, and c.1399C>G p. Pro467Ala), the red arrow represents mutation sites. B. 3D structural model of the wild-type and mutant residues with *RPE65* c.1399C>G p. Pro467Ala (black arrow).

**Additional file 5: Table S1**. Genotypes of the 57 patients from 50 families of Chinese origin reported previously.

**Additional file 6: Table S2**. Bioinformatic analysis results of the novel variants. Results of Fathmm can be found at: http://fathmm.biocompute.org.uk/fathmm-xf/cgi-bin/results.cgi?session=eb039a07-6c14-4e0d-97e3-5d2bfe729b4b, and http://fathmm.biocompute.org.uk/fathmm-xf/cgi-bin/results.cgi?session=5b257644-dd4b-4d56-b759-8c894fabd033.

## Data Availability

Please contact authors for data requests.

## Abbreviations

ACMG: American College of Medical Genetics
BCVA: Best corrected visual acuity testing
BSLP: Bone-spicule-like pigment deposits
CFT: Central foveal thickness
CME: Cystoid macular edema
ERG: Full-field electroretinography
FAP: Fundus albipunctatus
HGMD: Human Gene Mutation Database
IRD: Inherited retinal disease
LCA: Leber congenital amaurosis
NGS: Next-generation sequencing
OMIM: Online Mendelian Inheritance in Man
RP: Retinitis pigmentosa
SD-OCT: Spectral domain optical coherence tomography
WYD: White-yellow dots

## References

[1] den Hollander AI, Roepman R, Koenekoop RK, Cremers FP (2008). Leber congenital amaurosis: genes, proteins and disease mechanisms. Prog Retin Eye Res.

[2] Astuti GD, Bertelsen M, Preising MN, Ajmal M, Lorenz B, Faradz SM, Qamar R, Collin RW, Rosenberg T, Cremers FP (2016). Comprehensive genotyping reveals RPE65 as the most frequently mutated gene in Leber congenital amaurosis in Denmark. Eur J Hum Genet.

[3] Liu J, Bu J (2017). A gene scan study of RPE65 in Chinese patients with leber congenital amaurosis. Chin Med J.

[4] Wang H, Wang X, Zou X, Xu S, Li H, Soens ZT, Wang K, Li Y, Dong F, Chen R (2015). Comprehensive molecular diagnosis of a large Chinese leber congenital amaurosis cohort. Invest Ophthalmol Vis Sci.

[5] Chung DC, Bertelsen M, Lorenz B, Pennesi ME, Leroy BP, Hamel CP, Pierce E, Sallum J, Larsen M, Stieger K (2018). The natural history of inherited retinal dystrophy due to biallelic mutations in the RPE65 gene. Am J Ophthalmol.

[6] Hull S, Mukherjee R, Holder GE, Moore AT, Webster AR (2016). The clinical features of retinal disease due to a dominant mutation in RPE65. Mol Vis.

[7] Gao FJ, Li JK, Chen H, Hu FY, Zhang SH, Qi YH, Xu P, Wang DD, Wang LS, Chang Q (2019). Genetic and clinical findings in a large cohort of Chinese patients with suspected retinitis pigmentosa. Ophthalmology.

[8] Zhong Z, Rong F, Dai Y, Yibulayin A, Zeng L, Liao J, Wang L, Huang Z, Zhou Z, Chen J (2019). Seven novel variants expand the spectrum of RPE65-related Leber congenital amaurosis in the Chinese population. Mol Vis.

[9] Yang G, Liu Z, Xie S, Li C, Lv L, Zhang M, Zhao J (2017). Genetic and phenotypic characteristics of four Chinese families with fundus albipunctatus. Sci Rep.

[10] Jacobson SG, Cideciyan AV, Aleman TS, Sumaroka A, Schwartz SB, Windsor EA, Roman AJ, Heon E, Stone EM, Thompson DA (2007). RDH12 and RPE65, visual cycle genes causing leber congenital amaurosis, differ in disease expression. Invest Ophthalmol Vis Sci.

[11] Roman AJ, Cideciyan AV, Schwartz SB, Olivares MB, Heon E, Jacobson SG (2013). Intervisit variability of visual parameters in Leber congenital amaurosis caused by RPE65 mutations. Invest Ophthalmol Vis Sci.

[12] El Matri L, Ambresin A, Schorderet DF, Kawasaki A, Seeliger MW, Wenzel A, Arsenijevic Y, Borruat FX, Munier FL (2006). Phenotype of three consanguineous Tunisian families with early-onset retinal degeneration caused by an R91W homozygous mutation in the RPE65 gene. Graefe's Arch Clin Exp Ophthalmol.

[13] Li S, Xiao X, Yi Z, Sun W, Wang P, Zhang Q (2019). RPE65 mutation frequency and phenotypic variation according to exome sequencing in a tertiary centre for genetic eye diseases in China. Acta Ophthalmol.

[14] Philp AR, Jin M, Li S, Schindler EI, Iannaccone A, Lam BL, Weleber RG, Fishman GA, Jacobson SG, Mullins RF (2009). Predicting the pathogenicity of RPE65 mutations. Hum Mutat.

[15] Huang XF, Huang F, Wu KC, Wu J, Chen J, Pang CP, Lu F, Qu J, Jin ZB (2015). Genotype-phenotype correlation and mutation spectrum in a large cohort of patients with inherited retinal dystrophy revealed by next-generation sequencing. Genet Med.

[16] Thompson DA, Gyurus P, Fleischer LL, Bingham EL, McHenry CL, Apfelstedt-Sylla E, Zrenner E, Lorenz B, Richards JE, Jacobson SG (2000). Genetics and phenotypes of RPE65 mutations in inherited retinal degeneration. Invest Ophthalmol Vis Sci.

[17] Morimura H, Fishman GA, Grover SA, Fulton AB, Berson EL, Dryja TP (1998). Mutations in the RPE65 gene in patients with autosomal recessive retinitis pigmentosa or leber congenital amaurosis. Proc Natl Acad Sci USA.

[18] Takahashi Y, Chen Y, Moiseyev G, Ma JX (2006). Two point mutations of RPE65 from patients with retinal dystrophies decrease the stability of RPE65 protein and abolish its isomerohydrolase activity. J Biol Chem.

[19] Soens ZT, Branch J, Wu S, Yuan Z, Li Y, Li H, Wang K, Xu M, Rajan L, Motta FL (2017). Leveraging splice-affecting variant predictors and a minigene validation system to identify Mendelian disease-causing variants among exon-captured variants of uncertain significance. Hum Mutat.

[20] Li S, Hu J, Jin RJ, Aiyar A, Jacobson SG, Bok D, Jin M (2015). Temperature-sensitive retinoid isomerase activity of RPE65 mutants associated with Leber Congenital Amaurosis. J Biochem.

[21] Kondo H, Qin M, Mizota A, Kondo M, Hayashi H, Hayashi K, Oshima K, Tahira T, Hayashi K (2004). A homozygosity-based search for mutations in patients with autosomal recessive retinitis pigmentosa, using microsatellite markers. Invest Ophthalmol Vis Sci.

[22] Jacobson SG, Aleman TS, Cideciyan AV, Sumaroka A, Schwartz SB, Windsor EA, Traboulsi EI, Heon E, Pittler SJ, Milam AH (2005). Identifying photoreceptors in blind eyes caused by RPE65 mutations: prerequisite for human gene therapy success. Proc Natl Acad Sci USA.

[23] Hamel CP, Griffoin JM, Bazalgette C, Lasquellec L, Duval PA, Bareil C, Beaufrere L, Bonnet S, Eliaou C, Marlhens F (2000). Molecular genetics of pigmentary retinopathies: identification of mutations in CHM, RDS, RHO, RPE65, USH2A and XLRS1 genes. J Fr Ophtalmol.

[24] Mo G, Ding Q, Chen Z, Li Y, Yan M, Bu L, Song Y, Yin G (2014). A novel mutation in the RPE65 gene causing Leber congenital amaurosis and its transcriptional expression in vitro. PLoS ONE.

[25] Li L, Xiao X, Li S, Jia X, Wang P, Guo X, Jiao X, Zhang Q, Hejtmancik JF (2011). Detection of variants in 15 genes in 87 unrelated Chinese patients with Leber congenital amaurosis. PLoS ONE.

[26] Simovich MJ, Miller B, Ezzeldin H, Kirkland BT, McLeod G, Fulmer C, Nathans J, Jacobson SG, Pittler SJ (2001). Four novel mutations in the RPE65 gene in patients with Leber congenital amaurosis. Hum Mutat.

[27] Xu F, Dong Q, Liu L, Li H, Liang X, Jiang R, Sui R, Dong F (2012). Novel RPE65 mutations associated with Leber congenital amaurosis in Chinese patients. Mol Vis.

[28] Fu Q, Wang F, Wang H, Xu F, Zaneveld JE, Ren HN, Keser V, Lopez I, Tuan HF, Salvo JS (2013). Next-generation sequencing-based molecular diagnosis of a Chinese patient cohort with autosomal recessive retinitis pigmentosa. Invest Ophthalmol Vis Sci.

[29] Sitorus RS, Lorenz B, Preising MN (2003). Analysis of three genes in Leber congenital amaurosis in Indonesian patients. Vis Res.

[30] Zernant J, Kulm M, Dharmaraj S, den Hollander AI, Perrault I, Preising MN, Lorenz B, Kaplan J, Cremers FP, Maumenee I (2005). Genotyping microarray (disease chip) for Leber congenital amaurosis: detection of modifier alleles. Invest Ophthalmol Vis Sci.

[31] Stone EM (2007). Leber congenital amaurosis—a model for efficient genetic testing of heterogeneous disorders: LXIV Edward Jackson Memorial Lecture. Am J Ophthalmol.

