# Supplementary figures and images for: Frequency and phenotypic characteristics of RPE65 mutations in the Chinese population
Source: Orphanet J Rare Dis. 2021 Apr 13;16:174. doi: 10.1186/s13023-021-01807-3 (PMC8097799; doi:10.1186/s13023-021-01807-3)

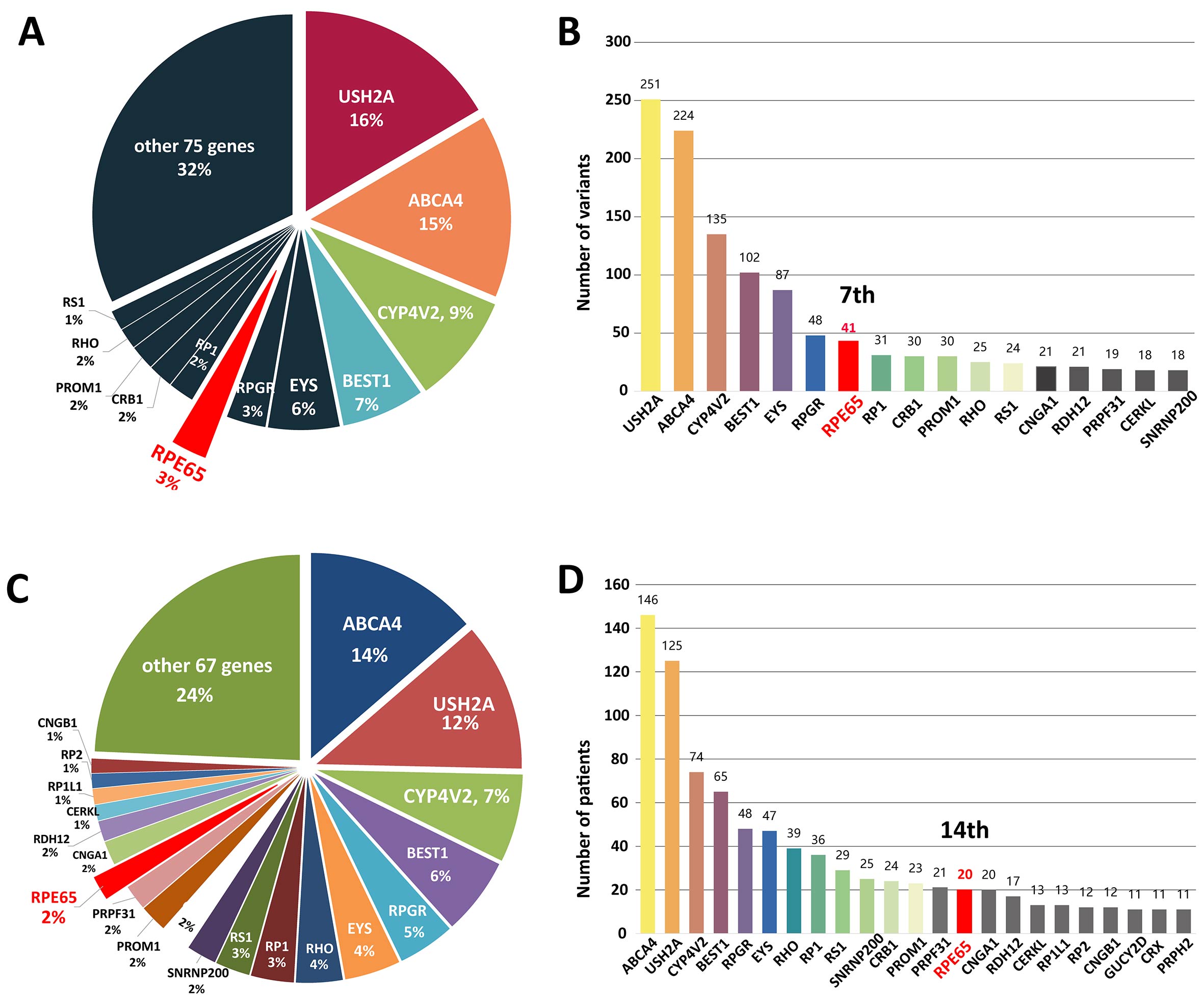

Supplement: Supplementary file 1 — Additional file 1: Figure S1. A. Distribution of inherited retinal disease (IRD)-causative genes variants (n = 1516) in 1069 patients. RPE65 mutations (n = 41) accounted for 2.70% of all the variants. B. Number of mutations of different IRD-causative genes. Only 41 variants were identified in the RPE65 gene, which ranked as the seventh most commonly detected gene in this cohort of patients with IRD. C. Percentage of IRD patients with variants in different causative genes; patients with RPE65 mutations only accounted for 1.87% of all patients (n = 1069). D. Number of IRD patients with different causative genes. Twenty patients with RPE65 mutations were identified, and they were placed as the 14th among all the patients. [file 13023_2021_1807_MOESM1_ESM.jpg]

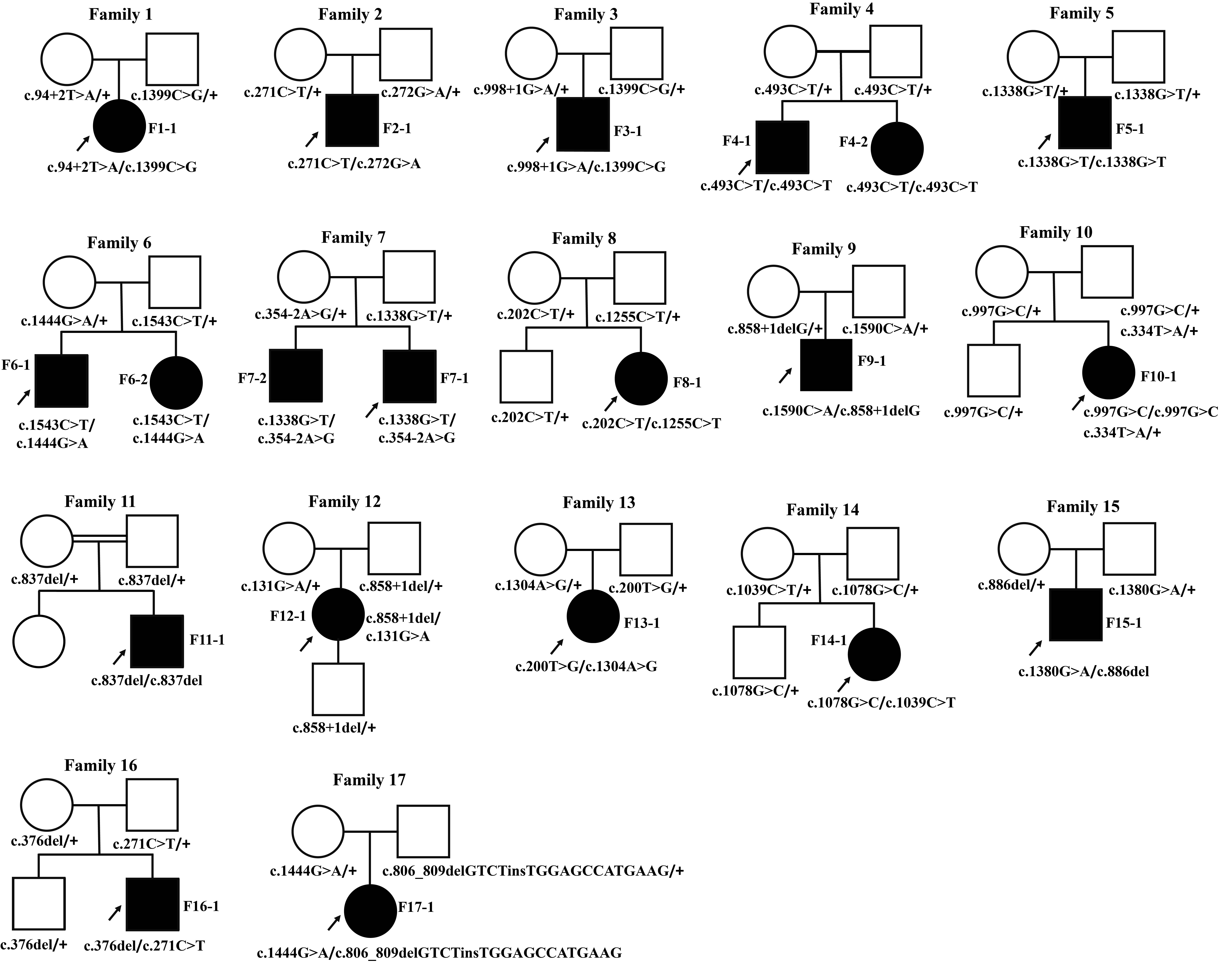

Supplement: Supplementary file 2 — Additional file 2: Figure S2. Pedigrees of the 17 families with RPE65 mutations. Filled symbols signify patients. Unfilled symbols represent unaffected family members. Arrows: probands. Square: male individuals; Circle: female individuals. A slash indicates a deceased person. [file 13023_2021_1807_MOESM2_ESM.jpg]

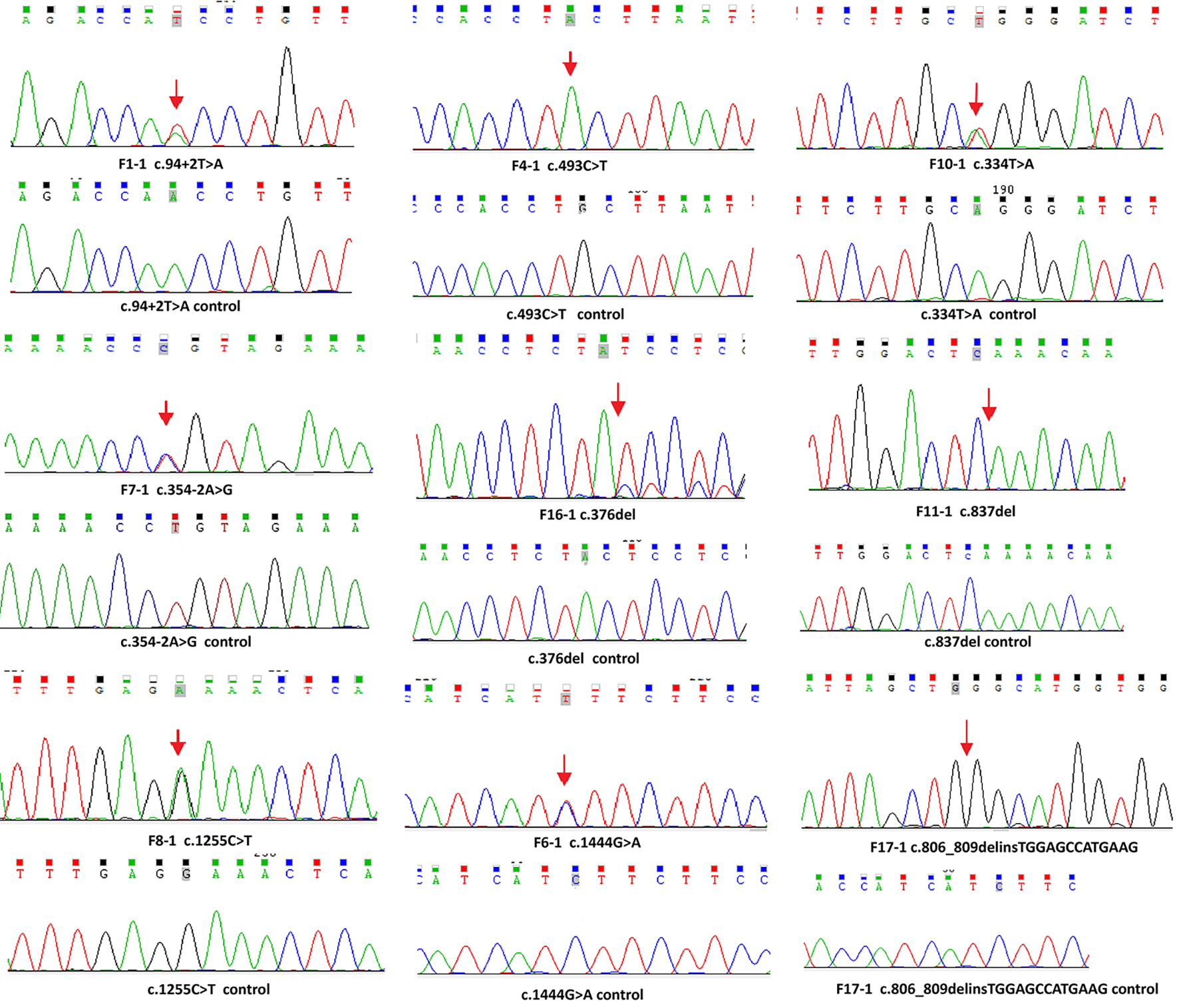

Supplement: Supplementary file 3 — Additional file 3: Figure S3. Sequencing results of the ten novel RPE65 variants identified in this study. Arrows indicate the position of the mutated nucleotide. Mutation c.806_809delinsTGGAGCCATGAAG, c.493C>T, c.354-2A>G, c.1255C>T, c.334T>A and c.1444G>A were shown in reverse strand. [file 13023_2021_1807_MOESM3_ESM.jpg]

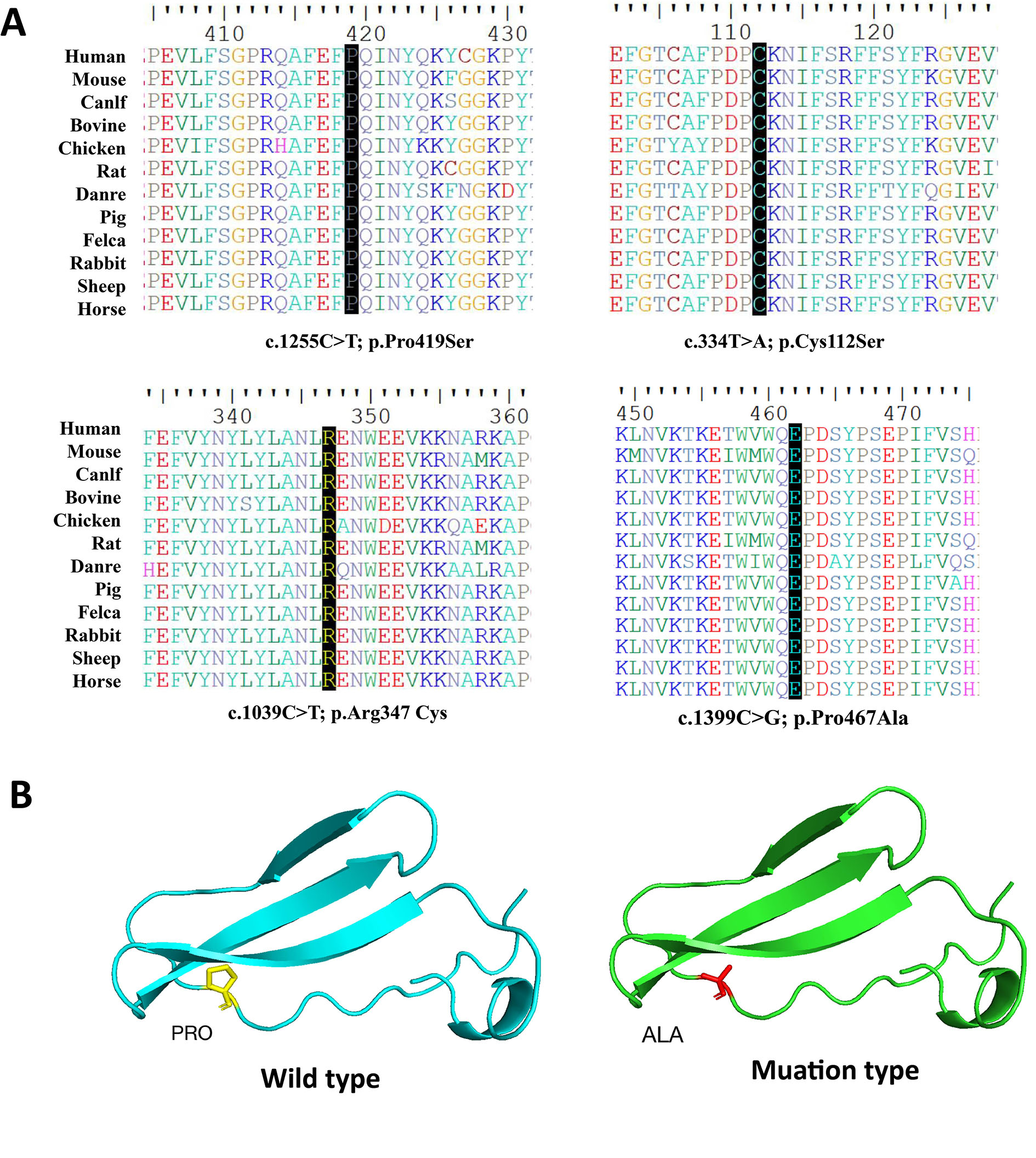

Supplement: Supplementary file 4 — Additional file 4: Figure S4. A. Multiple sequence alignment of different species of RPE65 mutations (c.1255C>T p.Pro419Ser, c.334T>A p.Cys112Ser, c.1039C>T p. Arg347Cys, and c.1399C>G p. Pro467Ala), the red arrow represents mutation sites. B. 3D structural model of the wild-type and mutant residues with RPE65 c.1399C>G p. Pro467Ala (black arrow). [file 13023_2021_1807_MOESM4_ESM.jpg]
