# Supplementary material for: Frequency and phenotypic characteristics of RPE65 mutations in the Chinese population
Source: Orphanet J Rare Dis. 2021 Apr 13;16:174. doi: 10.1186/s13023-021-01807-3 (PMC8097799; doi:10.1186/s13023-021-01807-3)
Supplement: Supplementary file 5 — Additional file 5: Table S1. Genotypes of the 57 patients from 50 families of Chinese origin reported previously. [file 13023_2021_1807_MOESM5_ESM.docx]

Supplementary Table 1. Genotype of the 37 patients from 35 families of Chinese origin reported previously.

| Reported literature | NO. of patients (families) | Mutations | Zygosity | Diagnosis |
| --- | --- | --- | --- | --- |
| Zhong et al., 2019[1] | 12 (12） | c.272G>A; p.Arg91Gln  c.1182dupT; p.Leu395SerfsX4 | Het  Het | LCA |
|  |  | c.493C>T; p.Gln165X | Hom | LCA |
|  |  | c.94G>T; p.Gly32Cys | Hom | LCA |
|  |  | c.272G>A; p.Arg91Gln  c.858+1delG | Het  Het | LCA |
|  |  | c.1338G>T; p.Arg446Ser | Hom | LCA |
|  |  | c.124C>T; p.Leu42Phe  c.130C>T; p.Arg44* | Het  Het | LCA |
|  |  | c.149T>C; p.Phe50Ser | Hom | LCA |
|  |  | c.130C>T; p.Arg44X | Hom | LCA |
|  |  | c.340A>C; p.Asn114His  c.425A>G; p.Asp142Gly | Het  Het | LCA |
|  |  | c.370C>T; p.Arg124* | Hom | LCA |
|  |  | c.130C>T; p.Arg44* | Hom | LCA |
|  |  | c.1399C>G; p.Pro467Ala  c.130C>T; p.Arg44* | Het  Het | LCA |
| Li et al., 2019[2] | 18 (15) | c.1301C>A; p.Ala434Glu  c.1399C>G; p.Pro467Ala | Het  Het | FAP |
|  |  | c.1301C>A; p.Ala434Glu  c.1399C>G; p.Pro467Ala | Het  Het | FAP |
|  |  | c.434C>A; p.Ala145Asp  c.1399C>G; p.Pro467Ala | Het  Het | FAP |
|  |  | c.434C>A; p.Ala145Asp  c.1399C>G; p.Pro467Ala | Het  Het | FAP |
|  |  | c.713C>G; p.Ser238Cys  c.1543C>T; p.Arg515Trp | Het  Het | FAP |
|  |  | c.713C>G; p.Ser238Cys  c.1543C>T; p.Arg515Trp | Het  Het | FAP |
|  |  | c.131G>A; p.Arg44Gln  c.1543C>T; p.Arg515Trp | Het  Het | FAP |
|  |  | c.722A>G; p.His241Arg  c.1067delA; p.Asn356Metfs*17 | Het  Het | LCA |
|  |  | c.271C>T; p.Arg91Trp  c.545A>G; p.His182Arg | Het  Het | LCA |
|  |  | c.1059dupG; p.Lys354Glufs*11 | Hom | LCA |
|  |  | c.271C>T; p.Arg91Trp  c.1451-1G>A | Het  Het | LCA |
|  |  | c.271C>T; p.Arg91Trp  c.1374G>A; p.Trp458* | Het  Het | LCA |
|  |  | c.271C>T; p.Arg91Trp  c.1450+1delG | Het  Het | LCA |
|  |  | c.94G>T; p.Gly32Cys  c.190C>G; p.Gln64Glu | Het  Het | RP |
|  |  | c.998+1G>A  c.1399C>G; p.Pro467Ala | Het  Het | RP |
|  |  | c.493C>T; p.Gln165*  c.999-1G>T | Het  Het | RP |
|  |  | c.825C>A; p.Tyr275*  c.1503T>G; p.Tyr501* | Het  Het | RP |
|  |  | c.639dupA; p.Ala214Serfs*20  c.1374G>A ;p.Trp458* | Het  Het | RP |
| Yang et al., 2017[3] | 1 (1) | c.639dupA; p.Ala214Serfs*20  c. 982C> T; p. Leu328Phe | Het  Het | FAP |
| Liu and Bu, 2017[4] | 1 (1) | c.1174A>C; p.Thr392Pro | Hom | LCA |
| Ge et al., 2015[5] | 1 (1) | c.310G>A; p.Gly104Ser  c.432C>G; p.Tyr144* | Het  Het | RP |
| Wang et al., 2014[6] | 1 (1) | c.1597T>A; p.Ser533Thr  c.89T>C; p.Val30Ala | Het  Het | RP |
| Chen et al. 2013[7] | 1 (1) | c.200T>G; p.Leu67Arg  c.430T>C; p.Tyr144His | Het  Het | LCA |
| Fu et al., 2013[8] | 1 (1) | c.200T>G; p.Leu67Arg  c.434C> A; p.Ala145Asp | Het  Het | RP |
| Xu et al., 2012[9] | 2 (1) | c.200T>G; p.Leu67Arg  c.1103A>G; p.Tyr368Cys | Het  Het | LCA |
|  |  | c.200T>G; Leu67Arg  c.1103A>G; p.Tyr368Cys | Het  Het | LCA |
| Li et al. 2011[10] | 1 (1) | c.997G>C; p.Gly333Arg  c.1059dupG; p.Lys354Glufs*11 | Het  Het | LCA |

Abbreviations: NO.: number; LCA: Leber congenital amaurosis; RP: retinitis pigmentosa; FAP: fundus albipunctatus.

1. Zhong Z, Rong F, Dai Y, Yibulayin A, Zeng L, Liao J, Wang L, Huang Z, Zhou Z, Chen J: **Seven novel variants expand the spectrum of RPE65-related Leber congenital amaurosis in the Chinese population**. *Molecular vision* 2019, **25**:204-214.

2. Li S, Xiao X, Yi Z, Sun W, Wang P, Zhang Q: **RPE65 mutation frequency and phenotypic variation according to exome sequencing in a tertiary centre for genetic eye diseases in China**. *Acta ophthalmologica* 2019.

3. Yang G, Liu Z, Xie S, Li C, Lv L, Zhang M, Zhao J: **Genetic and phenotypic characteristics of four Chinese families with fundus albipunctatus**. *Scientific reports* 2017, **7**:46285.

4. Liu J, Bu J: **A Gene Scan Study of RPE65 in Chinese Patients with Leber Congenital Amaurosis**. *Chinese medical journal* 2017, **130**(22):2709-2712.

5. Ge Z, Bowles K, Goetz K, Scholl HP, Wang F, Wang X, Xu S, Wang K, Wang H, Chen R: **NGS-based Molecular diagnosis of 105 eyeGENE((R)) probands with Retinitis Pigmentosa**. *Scientific reports* 2015, **5**:18287.

6. Wang J, Zhang VW, Feng Y, Tian X, Li FY, Truong C, Wang G, Chiang PW, Lewis RA, Wong LJ: **Dependable and efficient clinical utility of target capture-based deep sequencing in molecular diagnosis of retinitis pigmentosa**. *Investigative ophthalmology & visual science* 2014, **55**(10):6213-6223.

7. Chen Y, Zhang Q, Shen T, Xiao X, Li S, Guan L, Zhang J, Zhu Z, Yin Y, Wang P *et al*: **Comprehensive mutation analysis by whole-exome sequencing in 41 Chinese families with Leber congenital amaurosis**. *Investigative ophthalmology & visual science* 2013, **54**(6):4351-4357.

8. Fu Q, Wang F, Wang H, Xu F, Zaneveld JE, Ren H, Keser V, Lopez I, Tuan HF, Salvo JS *et al*: **Next-generation sequencing-based molecular diagnosis of a Chinese patient cohort with autosomal recessive retinitis pigmentosa**. *Investigative ophthalmology & visual science* 2013, **54**(6):4158-4166.

9. Xu F, Dong Q, Liu L, Li H, Liang X, Jiang R, Sui R, Dong F: **Novel RPE65 mutations associated with Leber congenital amaurosis in Chinese patients**. *Molecular vision* 2012, **18**:744-750.

10. Li L, Xiao X, Li S, Jia X, Wang P, Guo X, Jiao X, Zhang Q, Hejtmancik JF: **Detection of variants in 15 genes in 87 unrelated Chinese patients with Leber congenital amaurosis**. *PloS one* 2011, **6**(5):e19458.
