# Supplementary material for: Frequency and phenotypic characteristics of RPE65 mutations in the Chinese population
Source: Orphanet J Rare Dis. 2021 Apr 13;16:174. doi: 10.1186/s13023-021-01807-3 (PMC8097799; doi:10.1186/s13023-021-01807-3)
Supplement: Supplementary file 6 — Additional file 6: Table S2. Bioinformatic analysis results of the novel variants. Results of Fathmm can be found at: http://fathmm.biocompute.org.uk/fathmm-xf/cgi-bin/results.cgi?session=eb039a07-6c14-4e0d-97e3-5d2bfe729b4b, and http://fathmm.biocompute.org.uk/fathmm-xf/cgi-bin/results.cgi?session=5b257644-dd4b-4d56-b759-8c894fabd033. [file 13023_2021_1807_MOESM6_ESM.docx]

| Nucleotide Change | SIF | LRT | MutationTaster | FATHMM |
| --- | --- | --- | --- | --- |
| c.1444G>A | Damaging | Deleterious | Disease causing | Pathogenic |
| c.1255C>T | Damaging | Deleterious | Disease causing | Pathogenic |
| c.334T>A | Tolerated | Deleterious | Disease causing | Pathogenic |
| c.1039C>T | Damaging | Deleterious | Disease_causing | Pathogenic |
| c.94+2T>A | NA | NA | Disease_causing | Pathogenic |
| c.354-2A>G | NA | NA | Disease_causing | Pathogenic |
| c.376del | NA | NA | NA | NA |
| c.806_809delGTCTinsTGGAGCCATGAAG | NA | NA | NA | NA |
| c.837del | NA | NA | NA | NA |
| c.886del | NA | NA | NA | NA |

Supplementary Table 2. Bioinformatics analysis results of the novel variants.

Results of Fathmm can be found at: http://fathmm.biocompute.org.uk/fathmm-xf/cgi-bin/results.cgi?session=eb039a07-6c14-4e0d-97e3-5d2bfe729b4b, and http://fathmm.biocompute.org.uk/fathmm-xf/cgi-bin/results.cgi?session=5b257644-dd4b-4d56-b759-8c894fabd033.
